# MicroRNAs define distinct human neuroblastoma cell phenotypes and regulate their differentiation and tumorigenicity

**DOI:** 10.1186/1471-2407-14-309

**Published:** 2014-05-02

**Authors:** Leleesha Samaraweera, Kathryn B Grandinetti, Ruojun Huang, Barbara A Spengler, Robert A Ross

**Affiliations:** 1 Albert Einstein College of Medicine, 1300, Morris Park Ave, Bronx, NY 10461, USA; 2 Genomics Institute of the Novartis Research Foundation, San Diego, CA, USA; 3 Edison, NJ, USA; 4 Fordham University, 441 E. Fordham Road, Bronx, NY 10458, USA

**Keywords:** Neuroblastoma, Differentiation, Tumorigenicity, MicroRNAs, miR-375, miR-124, N-myc, HuD

## Abstract

**Background:**

Neuroblastoma (NB) is the most common extracranial solid tumor in children. NB tumors and derived cell lines are phenotypically heterogeneous. Cell lines are classified by phenotype, each having distinct differentiation and tumorigenic properties. The neuroblastic phenotype is tumorigenic, has neuronal features and includes stem cells (I-cells) and neuronal cells (N-cells). The non-neuronal phenotype (S-cell) comprises cells that are non-tumorigenic with features of glial/smooth muscle precursor cells. This study identified miRNAs associated with each distinct cell phenotypes and investigated their role in regulating associated differentiation and tumorigenic properties.

**Methods:**

A miRNA microarray was performed on the three cell phenotypes and expression verified by qRT-PCR. miRNAs specific for certain cell phenotypes were modulated using miRNA inhibitors or stable transfection. Neuronal differentiation was induced by RA; non-neuronal differentiation by BrdU. Changes in tumorigenicity were assayed by soft agar colony forming ability. N-myc binding to miR-375 promoter was assayed by chromatin-immunoprecipitation.

**Results:**

Unsupervised hierarchical clustering of miRNA microarray data segregated neuroblastic and non-neuronal cell lines and showed that specific miRNAs define each phenotype. qRT-PCR validation confirmed that increased levels of miR-21, miR-221 and miR-335 are associated with the non-neuronal phenotype, whereas increased levels of miR-124 and miR-375 are exclusive to neuroblastic cells. Downregulation of miR-335 in non-neuronal cells modulates expression levels of HAND1 and JAG1, known modulators of neuronal differentiation. Overexpression of miR-124 in stem cells induces terminal neuronal differentiation with reduced malignancy. Expression of miR-375 is exclusive for N-myc-expressing neuroblastic cells and is regulated by N-myc. Moreover, miR-375 downregulates expression of the neuronal-specific RNA binding protein HuD.

**Conclusions:**

Thus, miRNAs define distinct NB cell phenotypes. Increased levels of miR-21, miR-221 and miR-335 characterize the non-neuronal, non-malignant phenotype and miR-335 maintains the non-neuronal features possibly by blocking neuronal differentiation. miR-124 induces terminal neuronal differentiation with reduction in malignancy. Data suggest N-myc inhibits neuronal differentiation of neuroblastic cells possibly by upregulating miR-375 which, in turn, suppresses HuD. As tumor differentiation state is highly predictive of patient survival, the involvement of these miRNAs with NB differentiation and tumorigenic state could be exploited in the development of novel therapeutic strategies for this enigmatic childhood cancer.

## Background

NB is the most common extracranial solid tumor in children. The outcome of patients has improved over the years and the estimated 5-year survival rate for non-high risk patients is 90%, whereas that for high-risk patients is 50% [1].

Amplification of the N-*myc* proto-oncogene and cellular heterogeneity are two key factors that influence patient survival. The three basic cell types in NB tumors and derived cell lines differ in their morphological, biochemical and tumorigenic properties — whereas N-type neuroblastic cells are mildly malignant and have neuronal characteristics, S-type cells are non-tumorigenic with features of non-neuronal (glial, melanocytic and smooth muscle) precursor cells. I-type cancer stem cells, which can differentiate into either N or S cells, express stem cell marker proteins and are highly tumorigenic [2-4]. Thus, the three basic cell phenotypes represent distinct differentiation states of NB with distinct tumorigenic properties. All three cell types are present in tumors [4]. Clinically, cellular heterogeneity is predictive of patient outcome - patients with stroma-poor tumors comprising undifferentiated neuroblasts are frequently fatal whereas stroma-rich tumors or those with differentiated ganglion cells show a better prognosis [5]. Therefore, one approach to controlling the malignant potential of this tumor involves exploiting its unique differentiation capacity.

MicroRNAs (miRNAs) are important regulators of gene expression and function and hence differentiation. A role for miRNAs in neuroblastoma has been extensively studied mainly focusing on their association with respect to N-*myc* amplification, chromosomal imbalances, prognosis and retinoic acid (RA)-induced differentiation as discussed in four reviews [6-9]. These studies have revealed that large scale chromosomal imbalances result in dysregulated miRNAs which have a functional role in neuroblastoma pathogenesis and tumorigenicity. MiRNAs associated with N-*myc* amplification such as miR-17-92 cluster members are shown to be associated with NB tumorigenicity. Also, miRNAs associated with RA-induced differentiation of NB has been extensively studied as RA is used clinically in treating NB patients. These studies, as reviewed by Stalling et al., indicate that miRNA and DNA methylation changes following RA-treatment play a critical role in NB differentiation [9]. miRNAs modulated upon RA-treatment are shown to regulate key genes involved in differentiation, survival and tumorigenic properties of NB [9].

The present study is mainly focused on investigating the association of miRNAs with respect to the different cell phenotypes derived from NB and their role in regulating their intrinsic differentiation and tumorigenic properties with use of large panel of NB cell lines.

## Methods

### Cell culture and differentiation

The thirteen different human NB cell lines or clones, established from 8 patients’ tumors or bone marrow aspirates, used for these studies have been published previously (4). Seven cell lines or clones were isolated at Memorial Sloan-Kettering Cancer Center or Fordham University [SH-SY5Y, SH-EP1, BE(1)n, BE(2)-M17V, BE(2)-C, SK-N-LD, and SK-N-HM], three [SMS-KCN, SMS-LHN, and CB-JMN] were obtained from Dr. C. Patrick Reynolds (Texas Tech University Health Sciences Center) and SMS-KCN subsequently cloned [KCN-83n and KCNs], and one cell line, LA-N-1, was obtained from Dr. Robert C. Seeger (Children’s Hospital of Los Angeles) and cloned [LA1-55n and LA1-5s]. All cell lines were maintained in a 1:1 mixture of Eagle’s Minimum Essential Medium with non-essential amino acids and Ham’s Nutrient Mixture F12 (Invitrogen Corporation, Carlsbad, CA), supplemented with 10% fetal bovine serum (Hyclone, Logan, UT) without antibiotics.

### miRNA microarray

miRNAs were isolated using the miRVana miRNA isolation kit from Ambion (Austin, TX). Processing and initial microarray analysis of miRNA expression levels was done by LC Sciences (Houston, TX). Levels of 313 different miRNAs were assayed by these arrays. Three groups of miRNAs were deleted prior to analysis: i) miRNAs whose expression was barely detectable in all samples (i.e., with a mean fluorescence ≤ 100); ii) those with statistically non-significant differences (p ≥ 0.05) between (N and I) and Mix; and iii) data from hybridizations to the complementary strand of the miRNAs (S-hsa-miRNAs). The expression levels of miRNAs in different groups were analyzed by Student’s t-test.

### Clustering analysis

Unsupervised clustering based on miRNA expression profiles was generated using MultiExperiment Viewer (MeV) version 4 ( http://www.tm4.org/mev.html) using a complete linkage-clustering algorithm with a Spearman rank correlation metric.

### Semi-quantitative RT-PCR

Semi-quantitative RT-PCR was performed using the mirVana RT-PCR miRNA Detection Kit (Ambion, Austin, TX).

### qRT-PCR

cDNAs for miRNAs were synthesized using the TaqMan® MicroRNA Reverse Transcription kit and miRNA-specific primers and quantified using TaqMan assays (Applied Biosystems, Foster City, CA) by comparative ΔΔCt method. Expression levels of miRNAs were normalized to U6 and expressed as a fold change compared to the levels of a standard sample of SH-SY5Y or LA1-5s.

### Generation of miR-124-overexpressing BE (2)-C clones

miR-124-overexpressing lentivirus was purchased from SBI Biosciences (Mountain View, CA). BE(2)-C cells were infected at a multiplicity of infection of 10 according to manufacturer’s instructions and cloned using cloning cylinders.

### ^3^H-norephinephrine uptake

Cells growing in multiwell plates were incubated in triplicate with 250 nM (1.2 μCi) ^3^H-norephinephrine (PerkinElmer, Waltham, MA) for 45 min, washed 2 times, and lysed [10]; radioactivity was measured by liquid scintillation spectrometry and normalized to cell number.

### Colony forming efficiency

Colony-forming efficiencies (CFE) in soft agar were measured as described previously [4] and determined in quadruplicate in three independent experiments.

### Chromatin immunoprecipitation (ChIP) assays

ChIP assays used the EZ ChIP™ Chromatin Immunoprecipitation Kit (Upstate Biological, Lake Placid, NY). Chromatin isolated from BE(2)-C cells was incubated with anti-N-myc rabbit polyclonal antibody (sc-791) (Santa Cruz Biotechnology, Santa Cruz, CA); mouse monoclonal anti-RNA polymerase II antibody (clone CTD4H8) (Upstate Biological, Lake Placid, NY); or rabbit anti-goat IgG (Chemicon International, Temecula, CA). The primer sets used for amplification are available upon request.

### Stable and transient transfections

Stable SH-SY5Y sense-N-*myc* and LA1-55n antisense N-*myc* transfectants have been described [11]. miRNA inhibitors for miR-375, miR-335, and control oligos (100 nM) (Ambion, Austin, TX) were transiently transfected into BE(2)-C or SH-EP1 cells for 48 hrs using Lipofectamine 2000 (Invitrogen Corp., Carlsbad, CA).

### Western blot analysis

Western blot analysis of proteins was performed as previously described [4]. Primary antibodies used were rabbit anti-N-myc [(C-19) (SC 791)] (Santa Cruz Biotechnology Inc., Santa Cruz, CA), human antisera against Hu proteins (a kind gift of Dr. Myrna Rosenfeld, University of Pennsylvania Medical School) and, as controls, mouse anti-actin [(AC-74) (076 K4762)] (Invitrogen Corp., Carlsbad, CA) and mouse anti-Hsp72/73 [(W27) (HSP01)] (EMD Chemicals Inc., Gibbstown, NJ).

## Results and discussion

### miRNAs define distinct NB cell phenotypes

**Figure 1:**
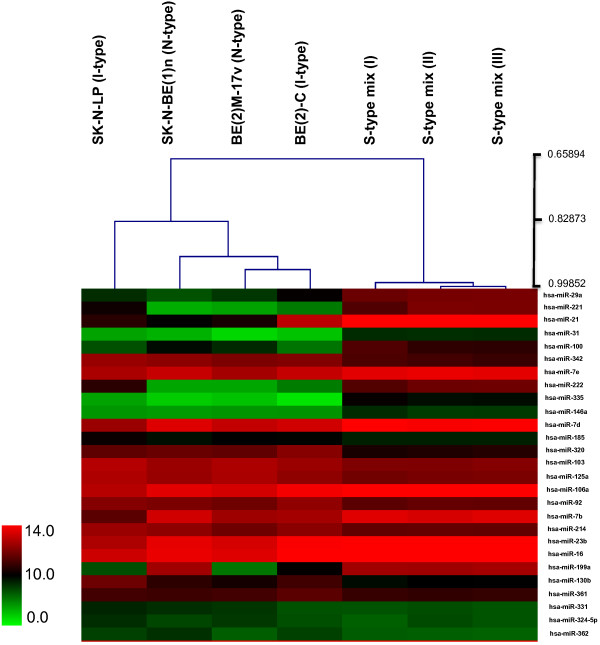
**Unsupervised clustering based on miRNA expression profiles was generated using MultiExperiment Viewer (MeV) version 4 (**http://www.tm4.org/mev.html**) and shows that N- and I-type cells are distinctly different from miRNA mix containing S-type cells.**

Levels of 313 different miRNAs from two N- and two I-type N-*myc*-amplified human NB cell lines were assessed in a miRNA microarray. These miRNA microarrays were performed as dual assays, where each sample and the control mix was hybridized to the same chip. The control mix included a mixture of miRNAs from these four cell lines with those from three different S-type cells. Inclusion of control mix enabled us to account for miRNAs that are expressed in neuroblastoma cells regardless of phenotype. As control mix also contains S-cell miRNAs, it also enabled us to identify miRNAs associated with the S-type non-tumorigenic cell phenotype by comparing it to neuroblastic cells (N- and I-type cells). Unsupervised hierarchical clustering analysis of miRNA microarray data shows that N- and I-cells cluster discretely from the S-cell miRNA-containing control mix (Figure 1). This is consistent with previous studies from our laboratory showing N- and I-cells share many characteristics not present in S-cells. N- and I-cells are tumorigenic and are neuroblastic in terms of their morphological and biochemical characteristics, whereas S-cells are non-tumorigenic and lack neuroblastic features [4]. Therefore, in the present study, we use the term "neuroblastic" to collectively define N- and I-cells and "non-neuronal" to define S-cells. Figure 1 shows representative miRNAs that are distinctly different between neuroblastic (N and I) cells and non-neuronal S-cells and either highly or poorly expressed in all NB phenotypes. MiRNAs that are distinctly different between the neuroblastic and the non-neuronal S-cells are of specific interest as they could regulate neuronal/non-neuronal differentiation and/or tumorigenicity. These were selected for further study.

To specifically identify miRNAs associated with the non-neuronal vs neuroblastic phenotype, miRNA levels were compared among cell lines representing these two phenotypes. The expression levels of twenty miRNAs that were highly significantly different between the two groups were ranked according to fold change (Additional file 1: Table S1). The majority of these miRNAs are highly expressed in the S-type cell containing mix and five out of top seven candidates that showed the highest fold change were selected for further analysis (miR-21, miR-31, miR-222, miR-221 and miR-335).

A second grouping compared expression levels between the two neuroblastic phenotypes (N vs I) to identify miRNAs that reflect the differences in neuronal maturation and/or malignant potential (Additional file 2: Table S2) as N-cells show more neuronal features and are less malignant than I-cells [4]. We also took into account published studies of miRNAs associated with neuronal differentiation in neuroblastoma. Three additional miRNAs, all showing higher expression in N compared to I cells— miR-124, miR-375 and miR-10b ― were selected for further analysis.

**Figure 2:**
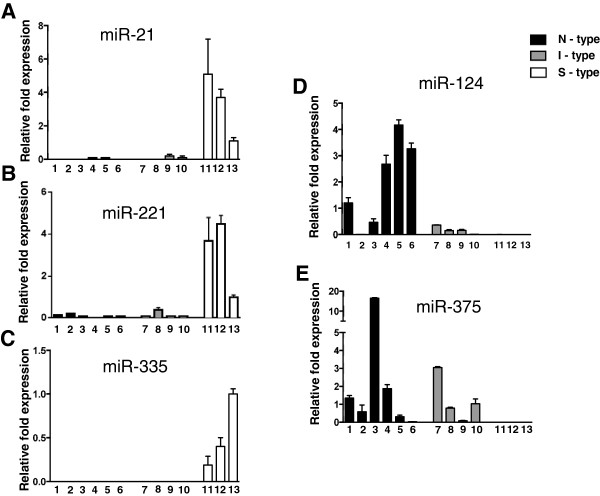
**qRT-PCR analysis of phenotype-specific miRNAs in NB cell lines.** The cell line panel includes six N-type [SH-SY5Y (1), SMS-LHN (2), BE(2)-M17V (3), LA1-55n (4), KCN-83n (5), SK-N-BE(1)n (6)]; four I-type [CB-JMN (7), BE(2)-C (8), SK-N-LD (9), SK-N-HM (10)]; and three S-type cell lines [SH-EP1 (11), SMS-KCNs (12), and LA1-5s (13)]. Levels of S-cell specific **(A)** miR-21, **(B)** -221 and **(C)** -335 were normalized to loading control U6 and expressed as a fold change compared to a standard sample of LA1-5s. Levels of **(D)** miR-124 and **(E)** -375 were normalized to loading control U6 and expressed as a fold change compared to a SH-SY5Y standard. Each bar represents the mean ± SEM of 3 or more samples.

Candidates from both analyses were validated by qRT-PCR using a panel of 13 human NB cell lines: six N-type, four I-type, and three S-type cell variants (Figure 2). qRT-PCR validation of five of the selected candidates confirmed the microarray expression pattern. Three miRNAs, miR-21, miR-221 and miR-335, show elevated expression in non-neuronal S cells and are barely detectable or very low levels in neuroblastic cells (Figure 2A, B, C). The expression of miR-21 and miR-221 is reported to play an oncogenic role in other types of cancers. However, their increased expression in non-tumorigenic S-type NB cells doesn't support a tumorigenic role for these miRNAs in NB, they could be involved in non-neuronal differentiation. Published studies of a role for the above two miRNAs in neuroblastoma have reported that elevated expression of miR-221 is correlated with N-myc amplification [12]. However, our observation of its higher levels of expression in S cells that have barely detectable levels of N-myc protein even in the presence of amplified N-*myc* gene [3] and its lower levels in neuroblastic cells those all express N-myc protein [4] doesn’t support its upregulation by N-myc. However, a study that analyzed miRNAs in 66 primary tumors and reported that higher levels of miR-21 correlated with favorable outcome of the patients supports our finding of its association with non-tumorigenic S-type cell [13].

Expression levels of two miRNAs, miR-124 and miR-375, were higher in the neuroblastic phenotype (Figure 2D and E). The six N-type cell lines have the highest levels of miR-124 expression [12.5-fold higher compared to I-type lines] suggesting its association with neuronal differentiation; S-cells have barely detectable levels of this miRNA. The second miRNA associated with a neuroblastic lineage, miR-375, is expressed at similar levels in both N- and I-cells while being barely detectable in S-type cells (Figure 2E).

### Drug-induced irreversible differentiation of I-type NB cancer stem cell confirms the association miRNAs with cell phenotype

**Figure 3:**
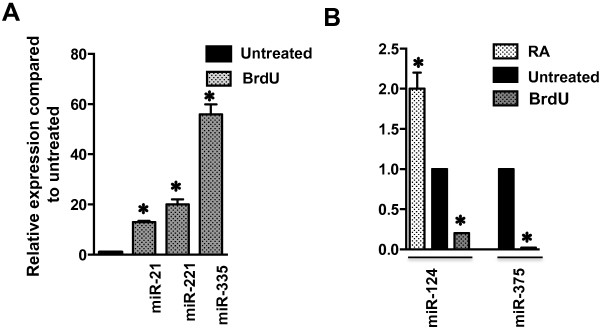
**Drug-induced irreversible differentiation of I-type NB cancer stem cell confirms the association miRNAs with cell phenotype. A**. Fold increases in miR-21, -221 and -335 in BE(2)-C cells differentiated to an S phenotype following a 2 week treatment with 10^-5^ M BrdU. **B**. Changes in miR-124 and -375 expression in BE(2)-C cells treated with RA or BrdU to induce an N or S phenotype, respectively. Each bar represents the mean ± SEM of 4–6 determinations normalized to untreated controls set =1.0.

Treatment of I-type NB stem cells with RA causes terminal neuronal differentiation whereas BrdU induces a non-neuronal S cell phenotype [4]. To confirm the association of the five miRNAs with cell phenotype, we analyzed their respective expression changes in I-type BE(2)-C cells differentiated by RA or BrdU. BrdU-induced S cell differentiation significantly increases expression of S-type-specific miRNAs - miR-21, miR-221 and miR-335 - by 13.0-, 20.0-, and 55.9-fold (P < 0.01), respectively (Figure 3A).

RA-induced differentiation increases miR-124 expression 2.0-fold (P < 0.01), whereas BrdU treatment causes a 5.0-fold reduction (P < 0.01) (Figure 3B). Similarly, miR-375 levels in I cells treated with BrdU decrease ~ 50-fold (P < 0.01) (Figure 3B). Thus, expression of these miRNAs characterizes the non-neuronal, non-tumorigenic NB cell phenotype.

### Functional role for miR-335 in S-cell phenotype

**Figure 4:**
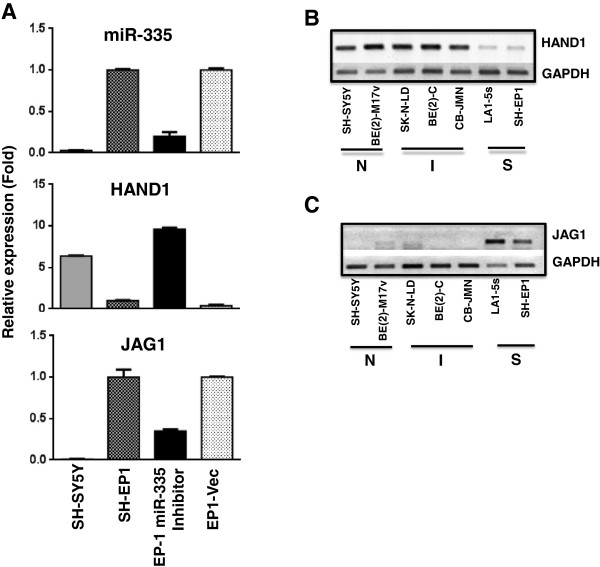
**MiR-335 regulates expression of HAND1 and JAG1- modulators of neuronal differentiation. A**. Quantitative changes in miR-335, HAND1, and JAG1 expression in miR-335 inhibitor-treated SH-EP1 cells. Each bar represents the mean ± SEM of three independent experiments. **B**. Semi-quantitative RT-PCR analysis of mRNA expression of HAND1 **(B)** and JAG1 **(C)** in cell lines of different phenotypes.

To investigate the role for miR-335 in non-neuronal cells, we down-regulated expression of this miRNA and measured expression of its predicted target genes and other genes that regulate cell differentiation. Short-term (4 day) down-regulation of miR-335 in S-type SH-EP1 cells did not result in any obvious morphological changes. However, reduction in miR-335 altered expression of key regulators of neuronal differentiation, HAND1 and JAG1. HAND1 levels, a proposed target of miR-335 (miRNA.org), increased upon suppression of miR-335 levels (Figure 4A). HAND1 is critical in differentiation of neural crest cells to catecholaminergic neurons [14]. Furthermore, neuroblastic cells that do not express miR-335 have the highest levels of HAND1 and non-neuronal S-cells that have the highest levels of miR-335 show least amount of HAND1 (Figure 2C and Figure 4B), suggesting miR-335 may play a critical role in NB differentiation. Down-regulation of miR-335 also decreases levels of JAG1, a known ligand for Notch 1 (Figure 4A). Down regulation of Notch signaling is instrumental for neuronal differentiation [15]. Accordingly, expression of JAG1 is highest in non-neuronal-S-cells and least in neuroblastic cells (Figure 4C). MiR-124, that is specific for neuroblastic cells, has been shown to decrease JAG1 expression leading to inactivation of Notch signaling during miR-124-induced neuronal differentiation [16]. Thus, reciprocal expression of miR-124 and miR-335 seems critical for NB differentiation. In addition, miR-335 is involved in inhibiting metastasis of NB [17], is transcriptionally repressed by N-myc, and has been shown to play a tumor suppressor role by directly targeting genes like TGF-β [18]. This finding suggests that miR-335 may also contribute to the non-tumorigenic properties of S-type cells. Previous reports accessing miRNAs in primary tumors showed that reduced levels of miR-335 expression are associated with favorable prognosis of patients [19,20], suggesting its potential use as both a prognostic and therapeutic agent.

### MiR-124 induces neuronal differentiation of I-type NB stem cells with concomitant reduction in malignant potential

**Figure 5:**
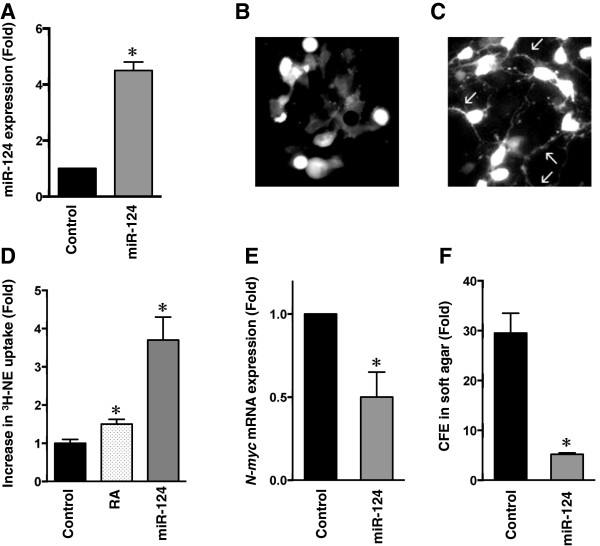
**miR-124 induces neuronal differentiation in I-type NB cells. A**. The miR-124 levels in control and miR-124-infected BE(2)-C. **B**. Immunofluorescence microscopy of BE(2)-C cells infected with a lentiviral-vector expressing GFP (control) or **(C)** BE(2)-C cells infected with lentiviral vector co-expressing miR-124 and GFP. Photomicrographs were taken two weeks after infection. Note the increase in number and size of multiple neuritic processes in **C** (arrows). **D**. ^3^H-NE uptake in BE(2)-C cells is increased 1.5–fold (P < 0.002) following treatment with RA and 3.7-fold (P < 0.001) with miR-124 lentiviral infection. **E**. *N*-*myc* mRNA levels are decreased ~2-fold (P < 0.008) in BE(2)-C/miR-124 lentiviral vector cells compared to control. **F**. Colony forming efficiencies (CFE) of BE(2)-C cells stably infected with miR-124 lentiviral vector or control. Note that CFE is reduced nearly 6-fold following infection (P < 0.001).

Our studies confirm the association of miR-124 to neuroblastic cell lines [21,22]. As miR-124 expression is higher in more neuronal N-cells (Figure 2D) and is elevated with RA-induced neuronal differentiation (Figure 3B), we sought to determine whether overexpression of miR-124 by itself is capable of inducing neuronal differentiation of tumorigenic I-type stem cells. Infection of I-type BE(2)-C cells with lentivirus coexpressing miR-124 and GFP induced a neuronal morphology within two weeks of infection. In BE(2)-C/miR-124-infected populations, miR-124 levels were 4.5-fold (P < 0.001) higher than BE(2)-C vector-infected populations (Figure 5A). GFP fluorescent cells expressing miR-124 have smaller, more rounded cell bodies and markedly increased numbers of elongated neurites (Figure 5C) compared to control cells (Figure 5B). Increased ^3^H-norephinephrine (^3^H-NE) uptake, an indicator of sympathetic neuron differentiation [23], was observed with both RA- and miR-124-induced BE(2)-C cells. RA treatment increased ^3^H-NE uptake by 1.5-fold (P < 0.002) and miR-124 infection increased it 3.7-fold (P < 0.001) (Figure 5D). RA-induced neuronal differentiation is known to reduce N-*myc* expression [24]. Likewise, miR-124-induced neuronal differentiation reduced N-*myc* mRNA levels nearly 2-fold (P < 0.008) (Figure 5E). Thus, increased expression of miR-124 induces neuronal differentiation in I-type stem cells.

13-*cis* retinoic acid treatment increases the survival of patients with high-risk NB [25]. Thus, we hypothesized that neuronal differentiation following miR-124 overexpression might also decrease cell tumorigenicity. Colony-forming efficiency (CFE) in soft agar revealed that, whereas control cells have a CFE of 29.5%, miR-124-infected BE(2)-C cells have a CFE of 5.2% (Figure 5F), a significant 5.7-fold reduction in malignant potential (P < 0.001).

Several other researchers have shown that miR-124 expression is related to neuronal differentiation [21,22]. Consistent with our findings, Le et al. showed that over expression of miR-124 in SH-SY5Y cells induces neurite outgrowth [26]. Clinically, neuronal differentiation in NB tumors is associated with reduced malignancy and tumor regression [25]. Therefore, miR-124 has the potential for use as a therapeutic miRNA in NB.

### N-myc regulates expression of miR-375

**Figure 6:**
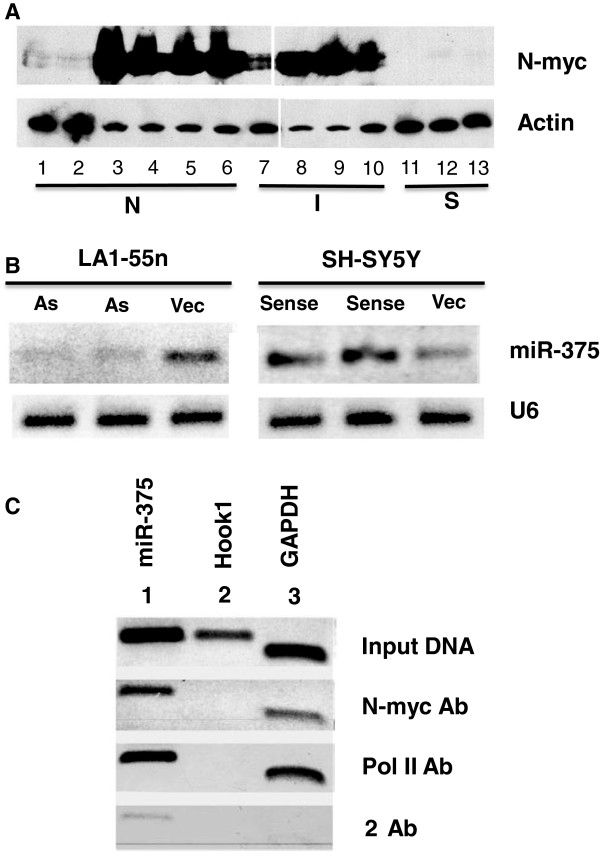
**miR-375 expression is regulated by N-myc. A**. Representative western blot of N-myc protein - SH-SY5Y (1), SMS-LHN (2), BE(2)-M17V (3), LA1-55n (4), KCN-83n (5), SK-N-BE(1)n (6), CB-JMN (7), BE(2)-C (8), SK-N-LD (9), SK-N-HM (10), SH-EP1 (11), SMS-KCNs (12), and LA1-5s (13). **B**. Changes in miR-375 levels, compared to U6, in N-myc antisense-transfected (As) LA1-55n cells and N-*myc* sense-transfected SH-SY5Y cells (sense) compared to vector-transfected (vec) controls. **C**. Chromatin immunoprecipitation analysis of N-*myc* regulation of miR-375. DNA-protein complexes cross-linked with formaldehyde were isolated and sonicated (Input DNA). Aliquots (1% of input) were immunoprecipitated with antibodies to N-myc or RNA polymerase II (Pol II) or with goat anti-rabbit secondary antibody. DNA was amplified by PCR with primers specific to *miR*-*375* E-box sequence (1), *Hook1* (2), and *GAPD* (3). Note that primers specific for miR-375 and GAPD E-box sequences gave a band indicating the association of N-myc with those genes.

Neuroblastic cells express both N-myc [3] and miR-375 (Figure 2B and Figure 5A). By contrast, S-cells have barely detectable levels of this proto-oncogene [3] or miR-375. miR-375 expression levels in N-*myc*-expressing cells are ~4-fold higher compared to non-expressing cells. Moreover, I-type stem cells differentiated to S-cells have barely-detectable levels of N-myc [3] and miR-375 (Figure 3A). Therefore, expression of miR-375 might be regulated by N-myc. This oncoprotein regulates gene expression by binding to E-box sequences (CACGTG) and the promoter region of the miR-375 gene contains several cis-acting elements, including two conserved non-canonical E-box sequences which are essential for optimal activity [27]. We measured changes in N-myc protein and miR-375 expression levels in clones of N-*myc* amplified LA1-55n N-cells stably transfected with an antisense construct to N-*myc*[28]. The >2-fold decrease in *N*-myc correlated with a 4-fold reduction in miR-375 (Figure 6B). Conversely, N-*myc* sense transfectants of N-*myc* non-amplified SH-SY5Y cells, which have a 1.8-fold increase in N-myc protein [28], have a 5-fold increase in expression of miR-375 (Figure 6B).

ChIP experiments confirmed that N-myc binds to one of two E-box sequences in the promoter region of the miR-375 gene (Figure 6C). N-myc binding specificity was confirmed with *GAPD* as a positive control [which contains a non-canonical E-box to which N-myc binds] [29] and *HOOK1* as a negative control (which lacks E-boxes). This experiment also shows that RNA Polymerase II is associated with the promoter region of miR-375 in BE(2)-C cells (Figure 6C).

### HuD is regulated by miR-375

**Figure 7:**
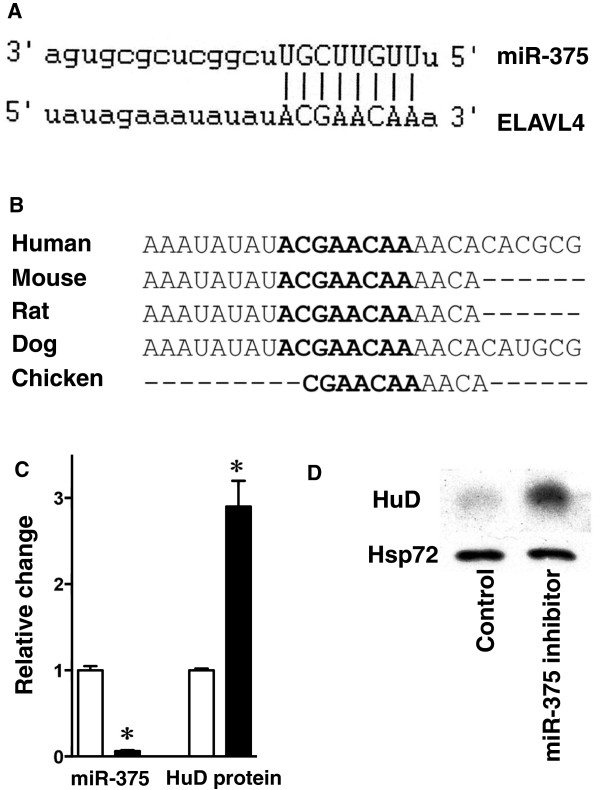
**MiR-375 regulates HUD. A**. ELAVL4 (HuD) mRNA 3’-UTR complement homology with miR-375 ( http://www.microRNA.org). **B**. Conservation of the miR-375 binding site in 3’-UTRs of ELAVL4 mRNA across different species. **C**. Relative levels of miR-375 and HuD protein in miR-375 inhibitor-treated BE(2)-C cells compared to control oligo-treated cells. Bars represent the mean ± SD of three independent experiments. **D**. Representative western blot of HuD protein levels in miR-375-inhibitor and control oligo-treated BE(2)-C cells.

We next screened target prediction sites for miR-375 target genes to identify possible partners involved with malignancy and differentiation in NB. Of interest, HuD, a neuronal-specific RNA-binding protein that influences neuronal differentiation [30], was among the predicted targets. The HuD 3’-UTR has a 7-mer miR-375 binding site (Figure 7A), which is highly conserved among species (Figure 7B). To determine whether this miRNA is involved in post-transcriptional regulation of HuD, BE(2)-C cells were transiently transfected with miR-375 inhibitor or negative control: miR-375 levels were reduced by ~95% (P < 0.05) and HuD protein levels increased 2.9-fold (P < 0.01) in inhibitor-treated cells compared to control (Figure 7C, D). Thus, miR-375 appears to down regulate HUD expression. A recent study showed that down regulation of HuD by miR-375 inhibits neuronal differentiation [30]. Therefore, in N- and I-type cells, high miR-375 levels may suppress neuronal differentiation by targeting HUD and thereby maintain the cells in a less differentiated, more proliferative, state. Supporting its role as a tumorigenic miRNA in neuroblastoma, increased expression of miR-375 is associated with patients with unfavorable outcome and metastatic dissemination [17] and miR-375 is one of the ten miRNAs whose increased expression is associated with advanced stage neuroblastoma [31].

## Conclusions

Our study shows that expression of specific miRNAs defines different NB cell phenotypes and are responsible for their associated tumorigenic and differentiation properties. The expression of three miRNAs, miR-21, miR-221 and miR-335, are exclusive to non-tumorigenic NB cell phenotype. Evidence suggests miR-335 maintains the non-neuronal features possibly by blocking neuronal differentiation. MiR-124 expression is exclusive to neuroblastic cells and overexpression of this miRNA in NB stem cells induces terminal differentiation with concomitant reduction in their malignant potential, suggesting a therapeutic potential for this miRNA in treating NB. The expression of miR-375 is associated with tumorigenic neuroblastic cell phenotype and we report that its expression is regulated by N-myc. MiR-375 downregulates HuD, a gene involved in neuronal differentiation. The differentiation state of the tumor is highly predictive of survival of NB patients. Thus, the involvement and association of these miRNAs in differentiation of NB could be used as prognostic markers and also in development of novel therapeutic strategies for this enigmatic childhood cancer.

## Competing interests

The authors declare that they have no competing interests.

## Authors’ contributions

LS and KG designed research and carried out experiments. RH performed experiments. LS, BS and RR analyze data and prepared the manuscript. All authors have read and approved the contents of final manuscript.

## Pre-publication history

The pre-publication history for this paper can be accessed here:

http://www.biomedcentral.com/1471-2407/14/309/prepub

## Supplementary Material

Additional file 1: Table S1 miRNAs with statistically significant differences in expression between neuroblastic (N + I) and non-neuronal lineage (S). Click here for file

Additional file 2: Table S2 miRNA expression as related to degree of neuronal differentiation. Click here for file
