# Supplementary material for: MicroRNAs define distinct human neuroblastoma cell phenotypes and regulate their differentiation and tumorigenicity
Source: BMC Cancer. 2014 May 2;14:309. doi: 10.1186/1471-2407-14-309 (PMC4038381; doi:10.1186/1471-2407-14-309)
Supplement: Additional file 1: Table S1 — miRNAs with statistically significant differences in expression between neuroblastic (N + I) and non-neuronal lineage (S). [file 1471-2407-14-309-S1.docx]

**Table S1: miRNAs with statistically significant differences in expression between neuroblastic (N + I) and non-neuronal lineage (S)**

| miRNA |  | T-test (group 1 vs group 2)^a^ | Group 1 (Ns and Is) | | | | Group 2 (miRNA mix including S cells)* | | | |
| --- | --- | --- | --- | --- | --- | --- | --- | --- | --- | --- |
|  | Fold change (Group 2 vs Group 1) |  | BE(2)-M17V | SK-N-BE(1)n | SK-N-LD | BE(2)-C | Mix repeat 1 | Mix repeat 2 | Mix repeat 3 | Mix repeat 4 |
| hsa-miR-335 | 154 | 0.0015 | 5 | 4 | 12 | 1 | 1,126 | 709 | 739 | 812 |
| hsa-miR-31 | 40 | 0.0004 | 3 | 8 | 12 | 5 | 312 | 286 | 310 | 223 |
| hsa-miR-146a | 14 | 0.0019 | 17 | 15 | 17 | 18 | 311 | 200 | 208 | 210 |
| hsa-miR-221 | 11 | 0.0003 | 12 | 9 | 1,210 | 37 | 2,485 | 4,071 | 3,914 | 3,475 |
| hsa-miR-29a | 8 | 0 | 234 | 111 | 306 | 1,094 | 3,278 | 3,761 | 3,841 | 3,371 |
| hsa-miR-222 | 7 | 0.0015 | 11 | 11 | 1,650 | 35 | 2,548 | 3,386 | 3,473 | 2,871 |
| hsa-miR-21 | 7 | 0.0004 | 1,343 | 987 | 1,607 | 7,837 | 19,405 | 21,659 | 19,919 | 18,583 |
| hsa-miR-100 | 6 | 0.0007 | 327 | 771 | 114 | 43 | 2,490 | 1,739 | 1,672 | 1,584 |
| hsa-let-7b | 2 | 0.0171 | 5,814 | 9,981 | 2,759 | 6,247 | 11,861 | 10,814 | 11,726 | 11,859 |
| hsa-let-7d | 2 | 0.0055 | 8,656 | 11,612 | 5,551 | 10,125 | 16,587 | 15,450 | 16,035 | 15,851 |
| hsa-let-7e | 2 | 0.0013 | 6,183 | 8,977 | 6,623 | 8,843 | 12,169 | 13,373 | 12,601 | 13,464 |
| hsa-let-7f | 2 | 0.0085 | 10,441 | 12,249 | 7,547 | 14,889 | 19,087 | 18,721 | 18,335 | 18,338 |
| hsa-let-7c | 2 | 0.018 | 10,531 | 12,640 | 6,864 | 15,779 | 18,621 | 18,226 | 17,783 | 18,163 |
| hsa-miR-106b | 1 | 0.014 | 3,450 | 3,954 | 4,399 | 4,982 | 2,809 | 3,111 | 3,014 | 2,838 |
| hsa-miR-93 | 1 | 0.0173 | 3,491 | 4,875 | 5,612 | 4,667 | 3,033 | 3,108 | 3,024 | 3,011 |
| hsa-miR-103 | 1 | 0.0064 | 6,790 | 5,633 | 7,359 | 5,536 | 4,117 | 4,140 | 4,417 | 3,835 |
| hsa-miR-107 | 1 | 0.0082 | 6,642 | 5,437 | 7,008 | 4,918 | 3,538 | 3,841 | 3,969 | 3,513 |
| hsa-miR-185 | 0 | 0.0059 | 996 | 698 | 1,179 | 929 | 431 | 446 | 450 | 372 |
| hsa-miR-342 | 0 | 0.0011 | 3,947 | 4,803 | 5,454 | 4,218 | 2,366 | 2,124 | 1,911 | 1,743 |
| hsa-miR-320 | 0 | 0.0059 | 2,972 | 3,244 | 2,932 | 4,515 | 1,367 | 1,442 | 1,551 | 1,310 |

**Notes**

^a^Student’s T-test was used to calculate the statistical significance between N + I (group 1) and mix (group 2); only miRNAs with p>0.02 were analyzed.

^b^miRNA mix contained miRNAs from N-type [BE(2)-M17V, SK-N-BE(1)n], I-type [BE(2)-C, SK-N-LD] and S-type [SH-EP1, SMS-KCNs, LA1-5s]
