# Supplementary material for: MicroRNAs define distinct human neuroblastoma cell phenotypes and regulate their differentiation and tumorigenicity
Source: BMC Cancer. 2014 May 2;14:309. doi: 10.1186/1471-2407-14-309 (PMC4038381; doi:10.1186/1471-2407-14-309)
Supplement: Additional file 2: Table S2 — miRNA expression as related to degree of neuronal differentiation. [file 1471-2407-14-309-S2.doc]

**Table S2: miRNA expression as related to degree of neuronal differentiation**

| microRNA | N-type cell lines | | I-type cell lines | | Mean | | Ratio^a^ |
| --- | --- | --- | --- | --- | --- | --- | --- |
|  | BE(2)-M17V | SK-BE(1)n | SK-N-LP | BE(2)-C | N lines | I lines | N/I |
| hsa-miR-383 | 11 | 760 | 24 | 5 | 386 | 15 | 26.58 |
| hsa-miR-369-3p | 3,340 | 23 | 9 | 192 | 1,682 | 101 | 16.72 |
| hsa-miR-10b | 3,651 | 2,304 | 166 | 215 | 2,977 | 191 | 15.63 |
| hsa-miR-375 | 12,992 | 22 | 273 | 752 | 6,507 | 512 | 12.70 |
| hsa-miR-376b | 2,492 | 12 | 14 | 203 | 1,252 | 108 | 11.56 |
| hsa-miR-98 | 1,171 | 2,308 | 356 | 44 | 1,740 | 200 | 8.70 |
| hsa-miR-100 | 327 | 771 | 114 | 43 | 549 | 78 | 7.03 |
| hsa-miR-7 | 9,261 | 901 | 1,125 | 410 | 5,081 | 768 | 6.62 |
| hsa-miR-124 | 162 | 932 | 123 | 63 | 547 | 93 | 5.86 |
| hsa-miR-199a | 6 | 893 | 46 | 107 | 450 | 77 | 5.85 |
| hsa-miR-199a* | 41 | 5,918 | 118 | 1,082 | 2,979 | 600 | 4.96 |

**Notes**

^a^ miRNAs were ranked by a ratio of N/I; only ratios >5.0 were analyzed. Note variability within groups.
